# Differential Expression of Non-Coding RNAs in Stem Cell Development and Therapeutics of Bone Disorders

**DOI:** 10.3390/cells12081159

**Published:** 2023-04-14

**Authors:** Anurag Mishra, Rishabh Kumar, Satya Narayan Mishra, Sivakumar Vijayaraghavalu, Neeraj Kumar Tiwari, Girish C. Shukla, Narasimman Gurusamy, Munish Kumar

**Affiliations:** 1 Department of Biochemistry, Faculty of Science, University of Allahabad, Prayagraj 211002, India; 2 Maa Gayatri College of Pharmacy, Dr. APJ Abdul Kalam Technical University, Prayagraj 211009, India; 3 Department of Life Sciences, Manipur University, Imphal 795003, India; 4 Department of IT—Satellite Centre, Babasaheb Bhimrao Ambedkar University, Lucknow 226025, India; 5 Department of Biological, Geological, and Environmental Sciences, 2121 Euclid Ave., Cleveland, OH 44115, USA; 6 Center for Gene Regulation in Health and Disease, 2121 Euclid Ave., Cleveland, OH 44115, USA; 7 Department of Pharmaceutical Sciences, College of Pharmacy, Nova Southeastern University, Fort Lauderdale, FL 33328, USA

**Keywords:** stem cells, lncRNA, miRNA, osteoblastogenesis, osteoclastogenesis, osteoporosis, osteoarthritis, and bone cancer

## Abstract

Stem cells’ self-renewal and multi-lineage differentiation are regulated by a complex network consisting of signaling factors, chromatin regulators, transcription factors, and non-coding RNAs (ncRNAs). Diverse role of ncRNAs in stem cell development and maintenance of bone homeostasis have been discovered recently. The ncRNAs, such as long non-coding RNAs, micro RNAs, circular RNAs, small interfering RNA, Piwi-interacting RNAs, etc., are not translated into proteins but act as essential epigenetic regulators in stem cells’ self-renewal and differentiation. Different signaling pathways are monitored efficiently by the differential expression of ncRNAs, which function as regulatory elements in determining the fate of stem cells. In addition, several species of ncRNAs could serve as potential molecular biomarkers in early diagnosis of bone diseases, including osteoporosis, osteoarthritis, and bone cancers, ultimately leading to the development of new therapeutic strategies. This review aims to explore the specific roles of ncRNAs and their effective molecular mechanisms in the growth and development of stem cells, and in the regulation of osteoblast and osteoclast activities. Furthermore, we focus on and explore the association of altered ncRNA expression with stem cells and bone turnover.

## 1. Introduction

Stem cells can develop into various cell types in the body during early stages of life and growth. In many tissues, they serve as an internal repair system, dividing essentially to replenish other cells, throughout the life of humans and other organisms. Upon division, each new cell has the potential to either remain a stem cell or become another type of cell with a more specialized function, such as a muscle cell, a red blood cell, or a brain cell. Stem cells are distinguished from other cell types by two important characteristics. First, they are unspecialized in nature, capable of renewing themselves through cell division, even after a long period of inactivity [1,2]. Previous studies suggest the potential role of non-coding (ncRNAs) in stem cell cycle regulation and developmental processes such as self-renewal, differentiation, and proliferation [3,4,5,6]. In last decades, the number of studies in ncRNAs has increased dramatically. ncRNAs are ribonucleic acid (RNA) molecules that are transcribed from DNA but not translated into protein. Sometimes ncRNAs are referred as RNA genes or functional RNA. Functionally important ncRNAs are transfer RNA (tRNA), ribosomal RNA (rRNA), small nuclear RNA (snRNA), small nucleolar RNA (snoRNA), micro RNA (miRNA), small interfering RNA (siRNA), extracellular RNA (exRNA), Piwi-interacting RNA (piRNA), and long non-coding RNA (lncRNA) [7]. The exact number of ncRNAs encoded within the human genome is unclear; however, recent studies suggest the existence of many thousands [8]. Different classes of ncRNAs participate in various cellular processes, e.g., RNA maturation (snRNA and snoRNA), gene expression and regulation (miRNA, piRNA, lncRNA, and circRNA), and protein synthesis (rRNA and tRNA) in eukaryotic cells [9]. The majority of the genome in both prokaryotes [10] and eukaryotes is transcribed into different classes of ncRNAs. According to the length, ncRNAs can be categorized into three categories: (i) a length of nucleotides less than 50 (miRNA, siRNA, and piRNA); (ii) a length of nucleotides ranging from 50 to 500 (rRNA and tRNA); and (iii) a length nucleotides greater than 200 (lncRNA and circRNA) [7,11]. A wide range of ncRNAs (miRNA, snRNA, snoRNA, piRNA, and lncRNA) control the growth and differentiation of stem cells and regulate stem-cell-mediated regeneration (cells and tissues) [3]. The ncRNAs interplay in epigenetic, transcription, and post-transcription mechanisms that determine the stem cell fate and improve the disease condition [12]. This review aims to explore the existing knowledge of ncRNAs in regulating the growth and development of stem cells with a special focus on the regulation of bone development and several bone disorders. Furthermore, this review will be helpful in designing potential treatment strategies for bone disorders in the near future. 

## 2. Stem Cells

Based on their source, potential to differentiate, and stage of development, there are four types of stem cells: embryonic stem cells (ESCs), adult stem cells, cord blood cells, and induced pluripotent stem cells. An embryonic stem cell is derived from the inner cell mass of four- or five-day-old blastocytes capable of differentiating into all types of cells. Adult stem cells are not derived from embryonic tissue, are found in various organ systems (brain, bone marrow, skin, and blood), and maintain the homeostasis in which they exist. Unlike ESCs, adult stem cells are not pluripotent, meaning they cannot become every cell type in the human body. Adult stem cells differentiate to replenish dying cells and regenerate damaged tissues. In many adult tissues, such as bone marrow, muscle, and brain, stem cells divide asymmetrically, producing two cells: one cell is genetically identical (stem cell), and the other cell is involved in tissue repair and regeneration. Cord blood stem cells are present in the umbilical cord and placenta. The ability of stem cells to differentiate into cell types has opened new avenues of scientific investigation and potential therapies for a myriad of diseases [13,14,15].

In ESCs, bones originate from three distinct lineages: (i) somites (form axial skeleton), (ii) the lateral plate mesoderm (forms the limb skeleton), and (iii) the cranial neural crest (forms branchial arch, and craniofacial bones and cartilage). Mesenchymal stem cells (MSCs) present in the developing embryo where ossification occurs. There are two bone formation pathways: (i) intramembranous ossification in which bone rises directly within preexisting mesenchymal connective tissue, and (ii) endochondral ossification in which bone rises within hyaline cartilage, developed from mesenchyme). After birth, MSCs and skeleton stem cells (SSCs) are responsible for bone homeostasis in the body [16,17,18].

## 3. Stem Cells Regulation

The growth and development of stem cells is a complex process, where numerous signaling pathways interplay predictory roles. The Wnt signaling pathway is a key regulatory pathway that plays a crucial role in the self-renewal and differentiation of stem cells. Wnt signaling is controlled by a delicate balance between positive and negative regulators, while disruption induces cancer development [19]. The assessment of the pluripotent abilities of MSCs *via* Oct4 and Sox2, mRNA expression factors, linked with cell stemness, these highly expressed in (ESCs), affect cell proliferation and differentiation [20,21]. c-Myc is a regulatory factor in cell proliferation and metabolism. Moreover, c-Myc gene overexpression induces tumorogenesis [22,23]. p53 regulates c-Myc, Sox2, and Oct4 expression, and assists stem cells in an undifferentiated state [24,25]. Pluripotent stem cells (PSCs) have diverse roles in developing medicines and understanding the biological process of embryonic development and specific diseases. The PSCs’ functions (self-renewal and multi-lineage differentiation) are regulated by several growth factors, including LIF, FGF4, BMPs, chromatin regulators, transcription factors, signaling pathways, and ncRNAs. Transcription factors including Oct4, Sox2, Nanog, and Klf4 are significantly expressed by PSCs, and maintain stemness and pluripotency. Importantly, ncRNAs with more than 200 bp in length act as essential epigenetic regulators in stem cell pluripotency and its specific lineage. Hence, exploring the molecular mechanisms underlying the determination of PSCs’ fate is significant and will have potential applications [26,27,28].

## 4. ncRNAs in Stem Cells Growth and Development

Stem cell growth, development, and differentiation are dynamic processes regulated by the interactions between external signaling, epigenetic factors, and other molecules that regulate gene expression. The two classes of ncRNAs (lncRNAs and miRNAs) are potential regulators of stem cell function. MiRNAs including miR-132, miR-145, miR-128-3p, miR-204-5p, miR-342-5p, miR-1297, hsa-miR-302, miR-26b-5p, and miR-10a significantly regulate the function of stem cells. H19, AK141205, MEG3, Pnky, ANCR, TINCR, HULC, SNHG7, and EPB41L4A-AS1 are the lncRNAs that are involved in stem cell growth and differentiation [5,26]. 

### 4.1. lncRNAs and Stem Cell Pluripotency

LncRNA is a class of RNA molecules with more than 200 nucleotides. Recent studies indicate the involvement of lncRNAs in several processes, including genomic imprinting, chromosome silencing, chromosome modification, transcriptional interference, and transcriptional activation. The altered expression of lncRNAs may induce changes in related proteins and uncontrolled transcription, developing the risk for various diseases [29,30,31]. In a study, 51 lncRNAs were abnormally expressed in postmenopausal women with osteoporosis (OP). However, some lncRNAs participate in the pathological process of OP by regulating mRNA expression or osteoclast differentiation [32]. In the Notch signaling pathway, the regulatory effect of H19 (lncRNA) on the expression of delta-like ligand-1 (DLL1), delta-like ligand-3 (DLL3), delta-like ligand-4 (DLL4), Jagged-1 (JAG1) and Jagged-2 (JAG2), by regulating the expression of miRNAs (miR-17, miR-107, miR-27b, miR-106b, and miR-125a) downstream, enhanced the expression of bone morphogenetic protein-9 (BMP 9) and induced MSCs osteogenic differentiation [33]. 

The study on PSCs and ESCs expressed that 133 lncRNAs were upregulated and 104 lncRNAs were downregulated in PSCs and ESCs compared with human fibroblasts [34]. Many lncRNAs regulate pluripotent transcription factors, including Oct4, Sox2, and Nanog. In a recent study, multiple pluripotency-associated lncRNAs were identified and embedded in the chromatin regulatory network with RNA-Seq and RAT-Seq [35,36,37]. lncRNAs present in the nucleus interact with chromatin modification factors, RNA binding proteins, and/or transcription factors to regulate gene expression. Moreover, lncRNA Gm15055 may recruit PRC2 to maintain H3K27me3 levels on HOXA genes [38]. In one mechanism, lncPRESS1 acts with SIRT6, inhibiting SIRT6 attachment to chromatin and regulating the histone H3K56 and H3K9 promoter acetylation to protect hESC pluripotency [39]. Another study reports that lncRNA-ES1 (AK056826), lncRNA-ES3 (BC026300), and lncRNA-ES2 (EF565083) are diversely expressed in hESC nuclei, and bind near the TSS of the Oct4 and Nanog promoters to increase hESC pluripotency. LncRNA-ES1 and lncRNA-ES2 interact physically with SUZ12 and SOX2, and are expressed as a modular scaffold for SUZ12 (or PRC2) and SOX2 in hESCs. Furthermore, the SOX2 factor binds with lncRNAs to prevent the binding of other pluripotency-associated transcription factors. LncRNA Zeb2-NAT deficiency enhances reprogramming efficiency and maintains ESCs’ self-renewal and pluripotency. Thus, Zeb2-NAT may be treated as an early marker for pluripotency loss [35,40].

### 4.2. lncRNAs and Stem Cells Differentiation

LncRNA is transcribed from the different genomic regions, including exons, introns, intergenic, and others. At present, more than 30,000 lncRNAs are identified in humans and mice, while few are recognized for their functions [41]. The self-renewal and differentiation characteristics of PSCs are efficiently regulated by lncRNA. LncRNA also functions as an essential regulator in a variety of cellular processes, like chromatin remodeling, transcription, post-transcriptional modification, intracellular trafficking, metabolism, and differentiation [27,42,43]. However, lncRNA is an essential component of the three-dimensional genome structure that mediates the development of gene regulatory chromosome loops [44,45]. Such chromatin loops may bring distant enhancer elements near to the core promoter and induce optimal gene expression [46,47]. In an investigation, functional analysis of the lncRNA, Snhg14, abundantly expressed in both ESCs and PSCs, confirmed that Snhg14 is required to maintain stem cell pluripotency. Moreover, lncRNA Oplr16 (Oct4 promoter-interacting lncRNA 16) is another pluripotency-associated chromatin RNA factor that coordinates intrachromosomal looping and DNA methylation in the Oct4 promoter region. Wang et al. used CRIST-seq to identify another reprograming-associated lncRNA, Peblr20 (Pou5F1 enhancer binding lncRNA 20), which binds to the Oct4 enhancers. Thus, Peblr20 utilizes a novel trans-epigenetic RNA mechanism to control stem cell fate [48,49].

### 4.3. miRNAs and Bone Stem Cells Growth and Development

MiRNAs are small ncRNAs originating from the hairpin or double-stranded RNA precursor (introns and exons) by RNA polymerase II [50]. This was first discovered in 1993 by Lee and colleagues [51]. MiRNAs are the most abundant class of small ncRNAs, ubiquitously expressed in animals, plants, and viruses, indicating their evolutionary significance [52]. MiRNA binds to the 3′ untranslated regions (UTR) of target mRNA, activates its translation and regulates stability [53]. According to the miRBase database (v-22), the release added 48 new species, now containing 38,589 hairpin precursors and 48,860 mature miRNAs from 271 organisms, including humans, animals, plants, unicellular algae, and viruses. Specifically, 1917 annotated hairpin precursors and 2654 mature miRNAs have been identified in humans [54]. More than 60% of all protein-coding gene expression is regulated by miRNAs. Moreover, miRNAs are expressed in fundamental biological processes like proliferation, differentiation, survival, and apoptosis in many cell types [55,56]. However, altered miRNA expression may contribute to pathological conditions in humans including cardiovascular disease, cancer, psychiatric disease, autoimmune disease, and neurological disease [57,58,59,60]. MiRNAs regulate gene expression through different mechanisms and mediate gene silencing at the post-transcriptional level [55]. 

Recent evidence suggests that miRNAs also regulate stem cell function and development by targeting multiple cell-cycle-associated genes (e.g., cyclins, CDKs, and CDKIs) and coordinating with the stem cell cycle progression. However, the mechanism underlying miRNA-mediated stem cell regulation is still incompletely understood. Initially, miRNA’s role in stem cell development was observed in knockout mice lacking Dicer and DGCR8; these are the components of miRNA biogenesis [61,62]. Dicer-deficient mice expressed defective cell cycle progression [63]. DGCR8-deficient ESCs showed either delayed or reduced expression of differentiation markers, and delayed kinetics of cell cycle progression. Mostly, such ESCs (DGCR8-deficient) are arrested in the G1 stage of cell cycles. Here, the main function of the miRNA pathway is to promote the ESCs G1–S-phase transition in cell cycle progression. The similarity in the phenotype of DGCR8 and Dicer mutants confirms that Dicer in ESCs functions mainly in the miRNA pathway. In stem cells, the predominant function of miRNAs is the regulation of cell cycle progression during cell differentiation [61,64,65]. In a recent study on mice, the cloning and sequencing of miRNAs miR-290–295 cluster and miR-296 are specific to ESCs, and their levels decrease as the stem cells differentiate. Collectively, miR-290–295 and miR-296 maintain pluripotency and induce differentiation [66,67]. Further, miR-21 and miR-22 levels increase substantially in the induction of differentiation. Studies on hESCs also show that pluripotent stem cells have unique miRNAs whose levels decrease with differentiation [61,68]. The endogenous small interfering RNAs (endo-siRNAs) and the piRNAs are two new classes of sncRNAs. MiRNA and siRNA synthesis depends on the Dicer pathway, whereas piRNAs are synthesized from a long single-stranded precursor by Piwi proteins. PiRNAs are usually 26–31 nucleotides long. PiRNAs are essential for stem cell self-renewal because Piwi proteins present on piRNA are required for stem cell maintenance. Moreover, a piRNA derived from a sub-telomeric region in Drosophila melanogaster has been found to be associated with germline stem cell self-renewal [69,70]. The fields of stem cells and miRNAs have converged with the identification of several stem-cell-specific miRNAs. In mouse ESCs, mirtrons, and canonical and shRNA-derived miRNAs have been identified. MiRNAs seem to regulate stem cell fate by finetuning protein levels of various factors [61,71].

Moreover, miRNAs are engaged in the regulation of osteoblast and osteoclast activity. For instance, miR-140-3p may be involved in the regulation of osteoblast differentiation by the acting growth factor (TGF) β3 signaling pathway, whereas inhibiting miR-31 expression intercepts osteoclast activity [72,73]. The role of miRNA in osteogenesis is now emerging. MiR-125b and miR-26a prevent the differentiation of MSCs into osteoblasts. In one mechanism, miR-26a prevents osteogenic differentiation by inhibiting human adipose-tissue-derived stem cells (ADSCs) whereas the miR-125b mechanism is unknown. As key players in the complex interplay among diverse RNA species, miRNAs have been considered research hotspots for several years [74,75]. 

### 4.4. lncRNAs in Osteogenic Differentiation

In bone tissue formation, MSCs differentiate osteogenically into osteoblasts, chondrocytes, and osteocytes. Such osteogenic differentiation stimulates ALP expression and calcium deposition, which is stimulated and regulated by several factors, including lncRNA. Deficiency in regulating factors may cause osteoporosis and osteogenesis imperfecta, particularly for elderly people and postmenopausal women. Hence, the understanding of the lncRNA regulatory contribution in osteogenesis may explore potential therapeutic targets for osteogenesis-deficient diseases [76,77].

**Figure 1:**
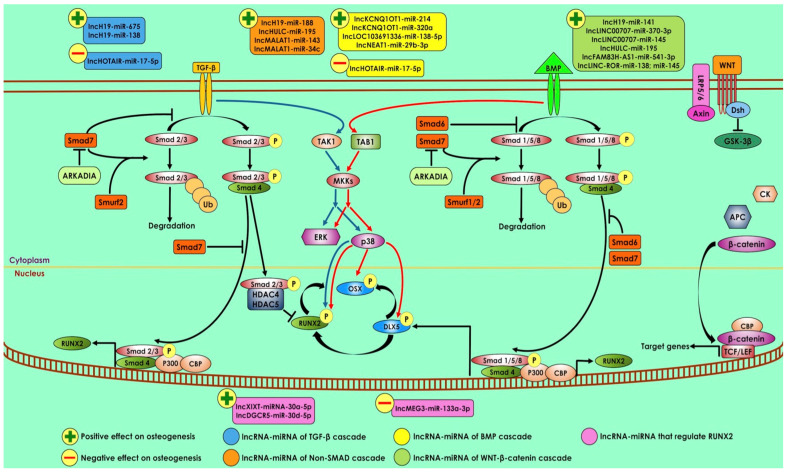
The contribution of various long non-coding RNAs and microRNAs in osteogenic pathways. Numerous lncRNAs and miRNAs have been shown to engage in different osteogenic pathways including BMP, transforming growth factor-β (TGF-β), and Wnt/β-catenin cascades, leading osteogenic differentiation in BMSCs. The binding between BMP and TGF-β with receptors activates SMAD-dependent and SMAD-independent cascades. SMAD2/3 (R-SMAD) is phosphorylated through the TGF-β SMAD-dependent signaling pathway. R-SMAD (phosphorylated) potentially interacts with SMAD4 and enters the nucleus. Further, R-SMAD and SMAD4 with CBP and P300 co-activators influence RUNX2 expression. Within the nucleus, R-SMAD interacts with HDAC4/5 and inhibits RUNX2 expression. Interestingly, non-phosphorylated R-SMAD is broken down by ubiquitination. Several ncRNAs including lncH19-miR-675 and lncH19-miR-675 positively regulate TGF-β SMAD-dependent pathways while lncHOTAIR-miR-17-5p regulates negatively. Significantly, Smurf1/2 and SMAD6/7 express inhibitory action in the BMP SMAD-dependent cascade (R-SMAD and SMAD1/5/8). Specifically, ncRNAs including lncKCNQ1OT1-miR-320a, lncKCNQ1OT1-miR-214, lncNEAT1-miR-29b-3p, and lncLOC103691336-miR138-5p are associated with BMSC growth and differentiation. Moreover, the SMAD-independent signaling event encourages the phosphorylation of RUNX2, DLX5, and OSX. The phosphorylation of RUNX2, DLX5, and OSX is significantly favored by lncHULC-miR-195, lncH19-miR-188, lncMALAT1-miR-143, and lncMALAT1-miR-34c axes. The Wnt/β-catenin pathway induces osteogenic differentiation in BMSCs through β-catenin transportation into the nucleus and following target gene expression. The Wnt/β-catenin pathway is positively regulated by lncLINC00707-miR-370-3p, lncH19-miR-141, lncHULC-miR-195, lncLINC00707-miR-145, lncLINC-ROR- miR-138, lncFAM83H-AS1-miR-541-3p, and miR-145. In RUNX2, lncXIXT-miRNA-30a-5p, and lncDGCR5-miR-30d-5p are the positive regulators while lncMEG3-miR-133a-3p inhibits RUNX2 expression.

In humans, MSCs osteogenesis is directed by the lncRNAs H19 and linc-ROR. H19 may be unregulated during osteogenic-related gene expression, and in vivo increases bone formation by targeting miR-22 and miR-141, which act as potent inhibitors of osteogenesis. Moreover, miR-22 and miR-141 may downregulate β-catenin expression, attenuating the Wnt/β-catenin signaling for osteoblastic activity [78]. Similarly, linc-ROR may also enhance the expression of osteogenic genes by targeting miR-138 and miR-145 [79]. Furthermore, the lncRNAs MEG3 and AK141205 may promote osteogenesis by dissociating SOX2 from the BMP4 promoter [80]. Osteogenesis of hADSCs downregulates expression of the lncRNA MIAT, whose deficiency encourages osteogenic differentiation in vitro and stimulates bone formation in vivo, indicating that MIAT inhibits osteogenesis whereas MIAT silencing reverses TNF-inhibited osteogenesis [81,82]. Further, osteogenesis of hBMSCs downregulates the expression of the lncRNA DANCR. Deficiency in DANCR expression increases ALP and osteogenic marker gene expression, promoting the cell cycle in the S phase, whereas overexpression of DANCR causes opposite effects [83]. Interestingly, lncRNA MIR31HG downregulation dramatically promotes osteogenesis and significantly reduces the osteogenic differentiation inhibition caused by hADSC inflammation. MIR31HG may also interact with NF-κB to inhibit bone formation. However, MIR31HG and NF-κB form a regulatory loop that improves osteogenesis in hADSCs under an inflammatory microenvironment. Given the importance of MIR31HG, it may be a therapeutic target for inhibiting inflammation and improving bone formation [84]. LncRNA ANCR silencing promotes osteoblast differentiation because it may interact with EZH2 to catalyze H3K27me3 in the runt-related transcription factor-2 (RUNX2) promoter and inhibit RUNX2 expression [85]. Osteogenic differentiation is regulated by lncRNAs such as H19 (lncH19), KCNQ1OT1, nuclear-enriched transcript 1 (NEAT1), metastasis-associated lung adenocarcinoma transcript 1 (MALAT1), lncRNA LINC00707, lncRNA HULC, lncRNA HOTAIR, maternally expressed gene 3 (lncRNA MEG3), XIXT, and DGCR5 in human bone marrow mesenchymal stem cells (hBMSCs) [86,87]. The lncRNA (AK141205) regulates the process of osteogenic differentiation of MSCs by upregulating CXCL13, while ANCR suppresses osteogenesis of periodontal ligament stem cells (PLSC) by sponging miR-758 [26,88,89]. The downregulation of MEG3 promotes osteogenic differentiation of human dental follicle stem cells by regulating the pathway Wnt/β-catenin [90]. The interaction of lncRNA and miRNA in several osteogenic signaling pathways, such as Wnt/β-catenin and TGF-β/BMP-SMAD-dependent and -independent pathways, is shown in Figure 1.

### 4.5. lncRNAs in Osteoclastogenesis

Osteoclastogenesis is a bone resorption process caused by the specialized cells called osteoclasts, developed by the myeloid progenitor [91]. Different mediators like RANKL, TGF-β1, and BMP are involved in the crosstalking between the osteoblast and osteoclast mechanisms, essential for bone health and bone metabolism [92]. Deregulation in osteoclast differentiation and activation is a sign of OP. LncRNA functions in distinct stages of osteoclast differentiation and maturation, such as monocytes to pre-osteoclasts, pre-osteoclasts to mature osteoclasts (bone resorption activity), and activation of mature osteoclasts (efficient bone resorption activity). Several lncRNAs are differently expressed in different phases of osteoclastogenesis, including lncRNA-4348, 4602, and 5840, in pre-osteoclasts, mature osteoclasts, and activated osteoclasts, respectively. Further exploration shows that 170 lncRNAs are identified as upregulated, and 348 lncRNAs are identified as downregulated in phases of osteoclastogenesis. Downregulation of lncRNA Gm12310 and Gm12308 are correlated with tumor necrosis factor, implicated in osteoclastogenesis [93,94]. In mice, the lncRNA AK077216 was shown to be significantly upregulated during osteoclastogenesis. In vitro, AK077216 promotes osteoclast differentiation and bone resorption in RAW264.7 cells. Importantly, AK077216 upregulates NFATc1, which acts as a key regulator in RANKL-induced osteoclast differentiation; this action is mediated through NIP45, which is repressed by lncRNA (AK077216) [95,96]. In the RAW264.7 cells model, Lee et al. revealed that lncRNA-Jak3 was identified as upregulated at three stages (pre-osteoclasts, mature osteoclasts, and activated osteoclasts) of osteoclast differentiation [97]. 

## 5. Exosomal ncRNAs and Bone Stem Cells 

Exosomes exist as membrane-bound extracellular vesicles synthesized inside the eukaryotic cells and act as a trans-regulatory element by transporting proteins. Exosomes contain biologically active molecules like DNA, RNAs (lncRNA, miRNA, circRNA, and tRNA), and some proteins, which are transferred to target cells. Exosomes communicate between the cells through endocytosis, ligand-receptor interactions, direct membrane fusion, or through signaling pathways [98,99]. Exosomes with low molecular weight, small size, stable structure, less toxicity, and other characteristics are employed as “nano-medicine carriers” in tissue regeneration and/or disease treatment [100,101]. Importantly, ncRNAs are present in exosomes that perform diverse functions including bone remodeling and bone-related disease [102]. BMSCs, osteoclasts, osteoblasts, lymphocytes, and macrophages secrete exosomes that regulate bone metabolism [103]. In bone metabolism, osteoblast differentiation is intercepted by exosomes secreted by osteoclasts [104]. However, the solicitation of osteoblast differentiation is regulated by exosomes produced by BMSCs and osteoblasts as well. In bone homeostasis, osteoblasts, osteoclasts, chondrocytes, and other cells secrete exosomal ncRNAs, which are involved in the regulation of bone-related diseases by inhibiting osteoclast differentiation, enhancing chondrocytosis, and promoting angiogenesis. Exosomal miRNAs secreted from MSCs exhibit potential regulatory effects on osteogenesis [102]. In humans, during BMSC osteogenic differentiation, the expression of exosomal miRNAs plays a regulatory role, and a low expression of miR-885-5p serves as a negative regulator by suppressing Wnt5 and RUNX2 [105]. 

### Exosomal miRNAs in Osteoblast and Osteoclast Differentiation

**Figure 2:**
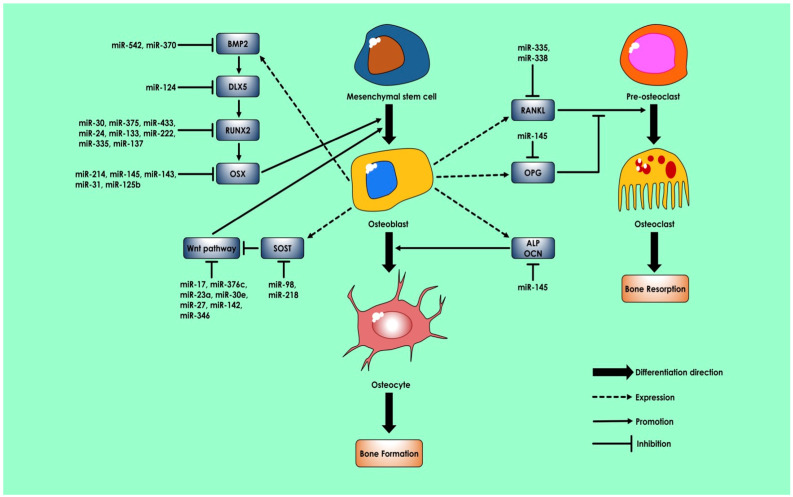
Regulation of bone formation by miRNAs. Various miRNAs play a regulatory role (activation or inhibition) in the differentiation of mesenchymal stem cells towards the formation of bone cells such as osteoblasts and osteocytes. The miRNAs regulate various molecules involved in signaling pathways, like Wnt, BMP2, DLX5, RUNX2, OSX, RANKL, SOST, and OPG, in osteogenesis (bone formation) and osteoclastogenesis (bone resorption).

Qin et al. determined how exosomes encouraged osteogenic differentiation, and miRNAs in exosomes (highly expressed: miR-27a, miR-206a, and miR196a) were identified, in which miR-196a showed more functional potential [106]. A disturbance between osteogenic differentiation and osteoclast differentiation causes OP; such a disturbance may be regulated by exosomal ncRNAs. In one study, exosomes containing miR-214 secreted by osteoclasts were transferred to osteoblasts by Ephrin-A2/Eph-A2 and inhibited osteoblast activity [107]. Interestingly, exosomal miR-214-3p inhibits osteoblasts, but this osteoblastic inhibition may reverse and promote bone formation, which may be a potential treatment for bone disease. Several studies reported that exosomal miR-30d-5p and miR-133b-3p might interfere with bone formation through the targeting gene RUNX2. However, miR-30d-5p and miR-133b-3p are highly expressed in the osteoblast-derived exosomal body (Figure 2) [102,108,109]. 

Moreover, osteoblast exosomes highly express miR-140-3p and inhibit the activity of osteoblasts [110]. In other studies, miRNA let-7 was observed in exosomes of mineralized osteoblasts and osteoblast precursors that promote osteogenesis through regulating the mobility of axis-like protein (AXIN-2) and the AThook 2 gene [111,112]. MiR-503-3p is expressed in osteoblast-derived exosomes and prevents osteoclast differentiation by inhibiting the gene receptor activator of nuclear factor kappa B (RANK) [113]. Several exosomal miRNAs secreted by mineralized osteoblasts, like miR-667-3p, miR-874-3p, miR-6769b-5p, miR-7044-5p, and miR-7668-3p, are highly expressed, and capable of enhancing the osteogenic differentiation of osteoblast precursors. This has been achieved through the inhibition of AXIN1 expression and the promotion of β-catenin expression [102,114].

During the early stage of osteogenic differentiation in hBMSCs, the expressions of exosomal miRNAs (miR-135b, miR-148a, miR-199b, and miR-218) increase significantly whereas miR-221 expression decreases. The increased expression of miR-135b, miR-148a, miR-199b, and miR218 is suggested to be involved as a regulator in bone formation in hBMSCs (Table 1) [102,105,115]. Additionally, in osteogenesis, several other miRNAs (miR-22, miR-27a, and miR-34a) have been identified in osteoblast-derived exosomes [115]. Therefore, exosomal miRNAs from osteocytic cells may modulate the differentiation of osteoblastic and osteoclastic activity, and inhibit or promote the bone formation process. In the management of bone disorders, like OP, OA, bone fractures, and several others, targeting exosomal miRNA therapy may be the core strategy [102,116].

## 6. Development of Bone Diseases

Bone tissue is a connective tissue where cells are arranged in rigid layers and intermingled with inorganic minerals. Bone cells called osteocytes are contained within a framework of an organic matrix, consisting of collagen and other proteins, which harden and strengthen the tissue. Bone diseases are a group of abnormal conditions that damage the skeleton system of the body and cause a bone to be weak and fragile, reduce bone remodeling, and accelerate bone to fractures. Low bone mineral density (BMD), OP, OA, gout, osteogenesis imperfecta, fibrous dysplasia, achondroplasia, Paget’s disease, and bone tumors are bone diseases characterized by progressive bone demineralization and damaged micro-architecture [117,118,119,120]. Genetic mutations, nutritional deficiencies, age, gender, body mass index (BMI), smoking, alcohol abuse, body weight, physical inactivity, and medications are significant inducers of bone disorders. OP is a disorder that is characterized by a loss of bone mass, which can lead to an increased risk of fractures [121,122]. OP accounts for about 200 million people worldwide; causing huge burdens of morbidity and mortality annually [123]. Osteogenesis imperfecta is a genetic disorder that is characterized by abnormal fragile bones [124]. Paget’s disease is a disorder that is characterized by the abnormal breakdown and formation of bone [125]. Further, bone cancer is characterized by uncontrolled growth of the bone cells, which is relatively rare. Tumors in other organs metastasize in the bone by the homing property of cancerous cells [126]. Moreover, the incidence of arthritis is reported as a major cause of disability [127]. Bone disorders can be distinguished into two main types: those that affect bone growth and those that involve damage or disease of the established bone [122]. 

## 7. ncRNAs and Bone Diseases

Several studies have reported an association between the abnormal expression of ncRNAs and the development of bone metabolic diseases. The key role of ncRNA in the progress of bone metabolic disease will assist in designing drugs for targeted therapy [128,129]. The interaction of important ncRNAs and their contribution to bone diseases are discussed below.

### 7.1. lncRNAs and SNPs in Bone Disease

Association of lncRNAs with single nucleotide polymorphism (SNP) in the coding and non-coding sequences of DNA is identified as a risk factor for the development of BMD and OP. SNP in the genomic region (1p36) has been reported as diversely linked with hip and spine BMD, and positively correlated with osteoporotic fractures [130,131]. In another study, Chen et al. reported that genomic variant rs6426749 (C/G) SNP at the 1p36.12 region was associated with lower BMD, and induced risk of OP. Genomic region 1p36.12 acts as an enhancer that regulates the expression of LINC00339, a lncRNA that plays a role in bone metabolism [132,133]. A study identified 26 specific loci in the genome that correspond to lncRNAs, efficiently associated with poor BMD and OP. In one investigation, SNP rs6894139 (T/G) in the lncRNA (MEF2C-AS1) was associated with femoral neck BMD, while SNP rs6465531 (G/A) in the lncRNA (LOC100506136) was linked with total hip BMD. Additionally, SNP rs1808124 (T/C) in BDNF-AS was associated with lower lumbar spine BMD [130].

### 7.2. circRNAs and Bone Diseases

Unlike linear RNAs, circRNAs do not possess 3′ and 5′ ends and are naturally expressed as closed-loop structures. CircRNAs are endogenous RNA transcripts having limited protein-coding efficiency. Over a period, circRNAs were irrelevant byproducts without any significant biological functions. Later, thousands of circRNAs and their biogenesis were discovered [134,135]. circRNAs regulate proliferation, differentiation, and apoptosis in several bone pathologies, including OP, osteoarthritis (OA), osteosarcoma, and lumbar intervertebral disc degeneration [136,137,138,139]. Many circRNAs are differentially expressed; they may accelerate or repress OP. In a recent investigation, circDNAH14 (circBase ID hsa_circ_0016624) prevented OP by the regulation of BMP-2 and miR-98 sponging [140]. In a clinical study, Liu et al. selected five samples for RNA sequencing among 40 postmenopausal osteoporosis patients (PMOP). A total of 250 differentially expressed circRNAs were estimated (64 circRNAs expressions were decreased, and 186 circRNAs were found to increase). circRNAs_0043813, 0001649, and 0005654 were upregulated, while circ_0007059, 0001204, and 0001795 were downregulated in the top six differentially expressed circRNAs. Further, Liu et al. examined circ_0007059 expression in osteoporotic samples, which were found to be reduced [141]. About 3938 upregulated and 1505 downregulated circRNAs were shown in osteoblast differentiation [142]. In OP, to prove the function of circRNA_0048211, Qiao et al. collected 60 samples (bone marrow) from PMOP; samples were cultured in an osteogenic induction medium. In the results, circRNA_0048211 protected OP by sponging miRNA-93-5p to regulate BMP-2 [143]. In osteogenesis regulation, circRNAs could promote osteogenesis through upregulating FOXO1 in OP [137]. Further, circRNAs including CDR1, CDK8, and SIPA1L1 are extensively implicated in osteogenesis differentiation [144].

In osteoclastogenesis, circRNAs are differentially expressed. In a study conducted on mature osteoclasts, 78 miRNAs and 38 circRNAs were found to be upregulated, while 24 miRNAs and 135 circRNAs were found to be downregulated [93]. Tumor necrosis factor-alpha (TNF-α) promotes bone resorption by osteoclast differentiation and inhibiting osteoblasts. Liu et al. examined the reduced level of circHmbox1 in TNF-α-induced osteoclast differentiation. However, circHmbox1 may inhibit RANKL-induced osteoclast differentiation by binding to miRNA-1247-5p. In OP, circRNAs have many miRNA-binding sites, function as miRNA sponges, and activate autophagy (osteoblast and osteoclast differentiation and proliferation) [145,146]. CircRNAs regulate several pathways, including the Wnt/β-catenin signaling pathway, BMP signaling pathway, and MAPK signaling pathway, which play a significant role in osteoporosis management. BMP2-induced osteogenesis was proved by the expression of circRNA_33287. This was upregulated in maxillary sinus membrane stem cells: circRNA_33287 downregulation inhibited several osteogenic biomarkers, such as RUNX2, ALP, and Osterix, while the upregulation exerted the opposite effect. However, circRNA_33287 is capable of inducing osteogenesis [144,147].

### 7.3. piRNAs and Bone Disease

Piwi-interacting RNAs (piRNAs) are a new subclass of ncRNAs that perform regulatory functions by explicitly interacting with Piwi proteins [148]. piRNAs play crucial roles in the differentiation, proliferation, and maintenance of mammalian germ cells [149,150]. piRNAs are also expressed in somatic cells (heart, brain, bone marrow, and other tissues), some of which eliminate mRNAs (post-transcriptional level), thereby affecting disease pathogenesis [148,151]. Piwi proteins guide piRNA to recognize and eliminate target mRNA [152]. In recent studies, piRNAs were expressed in the exosomes secreted by BMSCs. Exosomes are significantly expressed during the BMSCs’ osteogenic differentiation, indicating piRNAs’ contribution to osteogenesis [153,154]. PiR-63049 expression was shown to significantly increase in both bone tissues and plasma of PMOP and osteoporotic rats. Overexpression of piR-63049 could prevent osteoblastogenesis of BMSCs, while reduced piR-63049 expression could promote osteoblastogenesis by the Wnt2b/β-catenin signaling pathway. Additionally, in vivo knocking down of the expression of piR-63049 could attenuate bone loss by promoting bone formation. piRNA is also involved in tumor development: in patients with multiple myeloma the expression of piRNA-823 is upregulated [148,151]. The expression of piR-36741 is upregulated during the osteogenic differentiation of hBMSCs, while silencing of piR-36741 prominently suppresses osteogenic differentiation, resulting in reduced expression of osteogenic phenotype, osteoblast marker, and matrix mineralization. However, piR-36741 administration alleviated ovariectomy-induced osteoporosis in mice. Moreover, piR-36741 played a protective role in the osteogenic differentiation of BMSCs in mice with osteoporosis, where high expression reduced bone loss or demineralization [154,155].

### 7.4. siRNA and Bone Disease

Fire and colleagues discovered the silencing property of siRNAs in 1998 [156]. This has become an innovative approach to downregulating the expression of the target gene, particularly knocking down the gene in vitro or in vivo. siRNA is involved in several bone-specific pathways [157,158]. siRNAs express enormous potential as therapeutics in managing bone disorders including OP and bone cancer. In addition, the therapeutic approach of siRNA in bone disorders can be safe and efficient. In vivo delivery of siRNA to bone-specific cells is more challenging; however, various delivery systems such as polymer-mediated delivery, peptide-based delivery, lipid-based delivery, siRNA conjugate delivery, and delivery of therapeutic siRNA in cancer cells have been developed. However, a more efficient and cell-specific delivery system is needed [159]. Moreover, siRNA may have significant contributions to therapy. The potential ability of siRNAs is to knock down gene expression when the mRNA sequence is known. This may provide an inexpensive and efficient strategy for the management of a wide range of diseases. In bone regeneration, siRNA interferes with the expression of BMP inhibitors such as chordin and noggin, which manifests enhancing bone formation [160,161]. Different siRNAs target various regions of the same mRNA, with varying RNAi efficacies [162]. Almost 58–78% of siRNAs were observed to induce silencing of genes with >50% efficiency whereas only 11–18% of siRNAs induced 90–95% silencing [163]. To overcome siRNA delivery issues, various techniques have been developed to preserve and promote uptake by the target cells, and protect against enzymatic degradation within the cellular environment [164]. 

### 7.5. ncRNAs and Bone Cancer

The majority of cases of osteosarcoma (OS), a high-grade primary bone tumor, are found in teens and young adults. Pathologically, this illness is marked by spindle cells and aberrant osteoid development [165,166]. In human malignancies, long non-coding RNAs are typically expressed abnormally and support the growth, development, and spread of tumors [167,168,169,170]. As a result, they can be used as therapeutic, diagnostic, prognostic, and predictive biomarkers [171,172,173,174]. Numerous lncRNAs with either oncogenic or tumor-suppressive functions have been reported to have differential expression in osteosarcoma. In particular, 25,733 lncRNAs were found in research by Li et al., including 403 that were constitutively elevated in 34 pathways and 798 that were downregulated in 32 pathways (by a factor of two, *p* < 0.05) [175].

**Figure 3:**
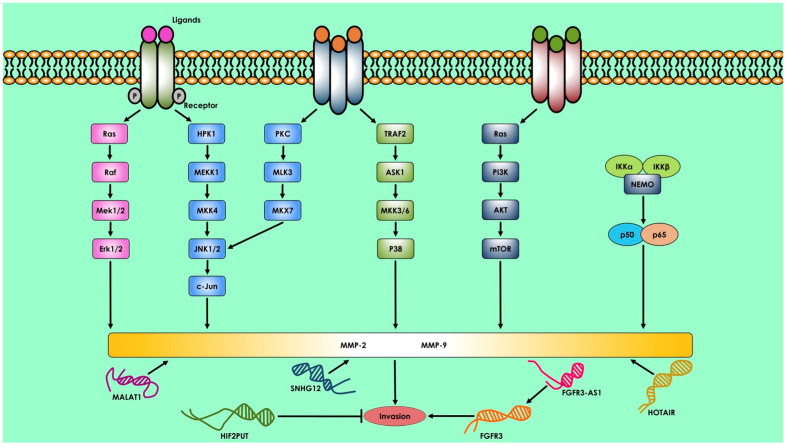
Long non-coding RNA that regulates osteosarcoma invasion and metastasis. MALAT1, SNHG12, HOTAIR, FGFR3-AS1, and HIF2PUT are lncRNAs that govern osteosarcoma invasion and metastasis. The Erk1/2, JNK1/2, P38, PI3K/Akt, and NF-B signaling pathways control MMP-2 and MMP-9 secretion. MMP-2 and MMP-9 govern osteosarcoma cell invasion.

Several lncRNAs are overexpressed in osteosarcoma, and one of them is metastasis-associated lung adenocarcinoma transcript 1 (MALAT-1), a lncRNA that controls the recruitment of pre-mRNA-splicing factors to transcription sites. The level of MALAT-1 expression is closely associated with the tumor’s propensity to metastasize. Dong et al. discovered in a different investigation that MALAT-1 promotes osteosarcoma cell proliferation, migration, invasion, and lung metastasis via the PI3K/Akt pathway (Figure 3) [176].

P50-associated COX-2 extragenic RNA (PACER), another lncRNA, is overexpressed in osteosarcoma cell lines and clinical tissues. In osteosarcoma, PACER has carcinogenic consequences by activating the COX-2 gene through the NF-B signaling cascade [177]. LncRNA MEG3 is underexpressed in several human malignancies, including non-small-cell lung cancer, colorectal cancer, and osteosarcoma. According to gain- and loss-of-function experiments, it is controlled by the lncRNA Ewing-sarcoma-associated transcript-1 (EWSAT1). When MEG3 is downregulated in the presence of EWSAT1, osteosarcoma cells grow, invade, and migrate. As a result, the advanced clinical stage (I/II vs. III) and the existence of distant metastasis are related to lower MEG3 expression in human osteosarcoma tissue [178,179,180,181].

Another highly upregulated liver cancer lncRNA-HULC was first discovered to have an oncogenic role in human hepatocellular carcinoma. Its gene, which has a transcript length of 500 bp and connects with ribosomes, is found in the chromosomal region 6p24.3 [182,183]. By lowering their expression, HULC works as a sponge for many miRNAs, including miR200a-3p, miR-9, and miR107 [184,185]. In hepatocellular carcinoma and colorectal carcinoma cell lines, it promotes tumor cell proliferation, invasion, and angiogenesis. In osteosarcoma cell lines and tissue samples, HULC is overexpressed. This overexpression is associated with an advanced clinical stage and a low rate of overall survival in osteosarcoma patients. In osteosarcoma cell lines, HULC inhibition decreases cell growth and invasion [186,187,188]. Breast, ovarian, lung, and hepatocellular carcinomas are among the malignancies in which HOTAIR is thought to be responsible for the pathogenesis [189,190,191,192]. By suppressing gene expression by histone H3K27 trimethylation and acting as a modular scaffold by binding PRC2 through the 5′ domain and LSD1/CoREST/REST complexes through the 3′ domain, it encourages the development and proliferation of tumor cells [193,194]. Although it is connected to other cancer forms, this molecular process in osteosarcoma is yet unclear. An intriguing case-control research in the Chinese population with 900 cases and 900 controls found that the HOTAIR gene variation rs7958904 was linked to a lower risk of osteosarcoma [195]. The lncRNA called HOXA transcript at the distal tip (HOTTIP) is overexpressed in osteosarcoma samples and is associated with an advanced clinical stage and a significant risk of metastasis [196]. In a number of malignant tumors, elevated expression of HOTTIP is linked to enhanced tumor cell proliferation, migration, and invasion [196,197,198]. It does so through controlling RNA-binding proteins, EMT-related molecules including E-cadherin, Snail1, Slug, and others, as well as HOXA genes like HOXA13. In osteosarcoma cell lines, HOTTIP knockdown prevents cell division, migration, and invasion [199,200,201].

The gene for taurine-upregulated gene 1 (TUG1), a 7.1-kb lncRNA, is found at chromosomal position 22q12.2 [202]. To promote osteosarcoma cancer, it appears to be stimulated by p53, interacts with polycomb repressive complex 2 (PRC2), and silences specific genes implicated in the G0/G1 cell cycle arrest [203]. In this situation, TUG1 functions as a miR-9-5p sponge and reduces POUF2F1 expression, indicating the existence of a competitive miR–lncRNA regulation network [204]. Additionally, it stimulates the growth of osteosarcoma tumors by upregulating EZH2 through miR-144-3p. Additionally, TUG1 knockdown inhibits Wnt/-catenin pathway activation, which is overridden by EZH2 overexpression [205]. It is interesting to note that osteosarcoma tissue clinical samples have high levels of TUG1 expression, while osteosarcoma cell line U2OS has impaired TUG1 expression, which slows cell growth and favors cell death [202]. In osteosarcoma tissue samples, TUG1 is overexpressed, and its overexpression is linked to a poor prognosis [206].

## 8. Therapeutics Approach of ncRNAs in Bone Disease

In modern medical science, ncRNAs may be the potential and efficient therapeutic option for targeting numerous bone disorders including osteoporosis, osteoarthritis, bone fractures, and bone tumors. Some important ncRNAs (lncRNA, miRNA, circRNA, piRNA, siRNA, and tRNA) and their role in managing bone disorders are discussed below.

### 8.1. lncRNAs in Osteoporosis and Osteoarthritis Treatment

**Figure 4:**
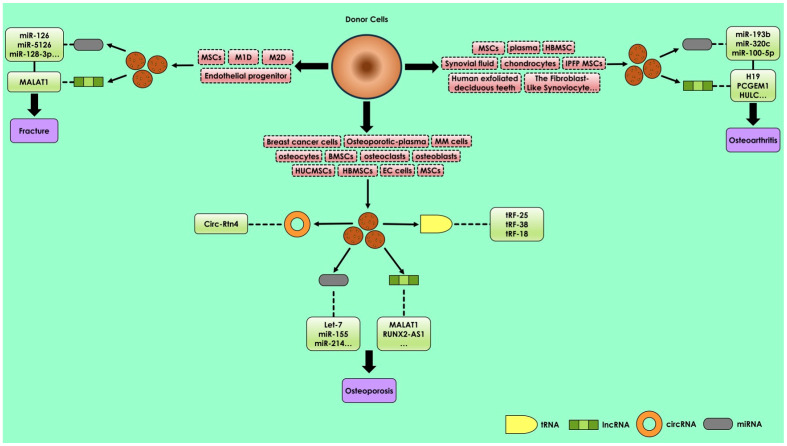
Regulatory action of various exosomal ncRNAs including lncRNA, miRNA, circRNA, and tRNA in osteoporosis, osteoarthritis, and bone fracture repairing. The expression of exosomal ncRNA may potentially modulate bone diseases. The major source cells that contribute exosomes, which participate in osteoporosis, are BMSCs, HUCMSCs, hBMSCs, MSCs, osteocytes, osteoclasts, osteoblasts, and osteoporotic plasma; in osteoarthritis are hBMSCs, MSCs, plasma, chondrocytes, and synovial fluids; and in bone fracture repairing are MSCs, M1D, M2D, and endothelial progenitors.

Bone fragility and risk of fractures are common in OP patients [207]. Huang et al. sequenced RNA (from femur subchondral tissues) and identified different gene expressions including 602 lncRNAs in patients with a femoral neck fracture and femoral head osteonecrosis. Further, data indicated that bone fractures were closely associated with specific lncRNAs. However, the differential expression of lncRNAs in fragility fractures compared with standard fractures needs to be explored [208]. lncRNAs are crucial for proper bone healing after a bone fracture by inflammation and angiogenesis [209]. lncRNAs also promote or suppress inflammatory pathways, implicated in bone homeostasis [210]. The role of lncRNAs in bone fractures is still unclear. Different lncRNAs such as H19, HOTAIR, and linc-p21 are identified as altered in the bone inflammatory state (rheumatoid arthritis and osteoarthritis) (Figure 4).

Further, H19, HOTAIR, and linc-p21 express potential targets for inflammatory modulation in osteoporotic patients [210,211]. LncRNA HOTAIR was significantly downregulated in synoviocytes of rheumatoid arthritis patients [212]. In rheumatoid arthritis (RA), HOTAIR overexpression reduced the secretion of IL-23 and IL-17, and decreased the number of pro-inflammatory cells (Th17), as well as diminishing levels of IL-1β, phospho-p65, and TNF-α in cartilage [213,214]. Moreover, lncRNA linc-p21 was found to be decreased in the blood of patients with rheumatoid arthritis, while its expression increased in human T cells [215]. Despite this, NKILA, ANRIL, and NEAT1 also regulate NF-kB signaling, a key player in inflammatory events, contributing to proper bone healing [216].

### 8.2. miRNAs in Bone Diseases and Fractures 

Healing of bone fractures is the proliferative process that facilitates bone repair fracture [217]. Initially, MSCs are recruited to the site of the fracture and differentiated as fibrocytes, osteoblasts, or chondrocytes, and these cells undergo several biomechanical stages whereby new bone is formed [218]. The aforementioned phases are potentially regulated by miRNAs. Overexpression of miR-214-5p was reported in patients suffering from intra-articular calcaneal and/or hand fractures, while its downregulation promoted osteoblastic cell viability and resisted apoptosis [219]. Using a mouse model with a femoral fracture, miR-186 activated the BMP signaling pathway to promote fracture healing by inhibiting *SMAD6* [220]. Another study showed the therapeutic impact of miR-29b-3p in femoral fracture repairing: an in vivo injection of miR-29b-3p improved healing in 14 days post-fracture [221]. The miRNAs miR-16, miR-25, miR-101, miR-19b-1, miR-92a, and miR-129-5p levels were shown to be dysregulated in bone fractures. Moreover, cells having miR-218 overexpression significantly promoted bone volume at 2 to 4 weeks post-fracture [222,223]. However, miRNAs regulate different cell functions but their effect in OP has not yet been well studied in well-characterized bone [123].

### 8.3. Treatment of Osteoporosis by Exosomal miRNAs

Exosomal miRNAs have significant roles in the pathological process and are a clinical marker for OP diagnosis. As shown in Table 1, exosomes containing miR-21 in osteoporosis patients interfere with osteogenesis events by the potential targeting of small mothers against decapentaplegic homolog-7 (SMAD7) [105]. In one study, MSC-derived exosomal miR-21 extracted from OP patients was expressed significantly higher than MSC-derived exosomal miR-21 extracted from healthy individuals [224]. Song et al. showed that inhibition in the levels of exosomal miR-155 secreted by vascular endothelial cells may reverse the inhibition of osteoclast differentiation and thereby prevent bone resorption. Hence, exosomal miR-155 may have the potential to be used in treating OP [225]. In a recent study, it was observed that exosomal miR-186 extracted from BMSCs could promote osteogenesis in OP postmenopausal women [226]. By injecting exosomal miR-151-5p in vivo, bone reduction may be prevented [227]. Therefore, the therapeutic impact of exosomal miRNAs in OP management has great potential. It is suggested that several miRNAs are highly expressed in exosomes, and increase or inhibit osteogenesis by regulating MSC differentiation. In OP treatments, signaling pathways, miRNAs, and related proteins have huge implications [228]. 

**Table 1:** Differential expression and regulation of exosomal ncRNAs in osteoporosis, osteoarthritis, and bone fracture repairing/healing by the different pathways.

Exosomal ncRNAs	Class of ncRNAs	Sources of Exosome	Differential Expression of ncRNAs	Types of Pathway	Mechanisms	Ref.
**In Osteoporosis**						
miR-186	miRNA	BMSCs	Increase	Hippo signaling	Promoting osteogenesis	[226]
lncRNA -H19	lncRNA	BMSCs	Increase	Angpt1/Tie2-NO signaling	Highly promoting osteogenesis and angiogenesis through mediating Angpt1/Tie2-NO signaling	[229]
hsa_circ_0006859	circRNA	Serum	Increase	miR-431-5p	Hsa_circ_0006859 suppressing osteoblastic differentiation and promoting adipogenic differentiation of hBMSCs	[230]
circ-Rtn4	circRNA	circ-Rtn4 modified BMSCs	N/A	miR-146a	Reducing the cytotoxicity and apoptosis of MC3T3-E1 cells induced by TNF-α	[231]
miR-1263	miRNA	HUCMSCs	Increase	Mob1	Inhibiting BMSCs apoptosis and preventing osteoporosis in rats	[232]
NONMMUT000375.2 NONMMUT071578.2	lncRNA	Osteoclasts	N/A	Genes related to osteoclast	Repressing the osteogenic differentiation of MC3T3-E1 cells	[233]
miR-29a	miRNA	hBMSCs exosome	Increase	Noggin	Promoting osteogenesis	[234]
miR-20a-5p	miRNA	Breast cancer cells (BCCs)	N/A	SRCIN1	Promoting the proliferation and differentiation of osteoclasts	[235]
miR-155	miRNA	Vascular endothelial cells	Increase	N/A	Inhibiting osteoclast induction	[225]
LncRNA MALAT1	lncRNA	BMSCs	N/A	miR-34c/ SATB2 axis	Promote osteoblast activity/enhance the activity of osteoblasts in osteoporotic mice	[236]
miR-31a-5p	miRNA	BMSCs	Increase	N/A	Promoting osteoclastogenesis and bone resorption	[237]
miR-21	miRNA	MSCs	Increase	SMAD7	Inhibition of osteogenic gene expression	[224]
lncRNA RUNX2-AS1	lncRNA	MM cells	N/A	RUNX2	Inhibiting the osteogenicity of MSCs	[238]
tRF-25 tRF-38 tRF-18	tRNA	Osteoporotic plasma Exosomes	Increase	N/A	Expressing good accuracy in the diagnosis of osteoporosis	[239]
miR-218	miRNA	Osteocytes	Decrease	Wnt signaling	Inhibited osteoblast differentiation	[240]
miR-151-5p	miRNA	BMSCs	N/A	N/A	Promoting osteogenic differentiation and protecting bone reduction	[227]
miR-214		Osteoclasts	Increase	EphrinA2/ EphA2	Inhibiting the function of osteoblasts	[107]
miR-214-3p	miRNA	Osteoclasts	Increase	N/A	Inhibiting osteoblast bone formation	[241]
miR-7044-5 pmiR-7668-3p miR-874-3p miR-667-3p miR-6769b-5p	miRNA and piRNA	Mineralized osteoblasts	Increase	AXIN1 β-catenin	Promoting the osteogenic differentiation of osteoblast precursors	[114]
miR-140-3p	miRNA	Osteoblasts	N/A	BMP2	Inhibiting the formation of osteoblasts	[110]
Let-7 AXIN2	miRNA	Osteoblast precursors/mineralized osteoblasts	Increase	HMGA2	Promoting osteogenesis	[111,112]
miR-503-3p	miRNA	Osteoblast	N/A	RANK	Preventing osteoclast differentiation	[113]
miR-218 miR-148a miR-199b	miRNA	hBMSCs	Increase or Decrease	N/A	Increased/decreased significantly during the early stage of osteogenic differentiation of hBMSCs	[105]
miR-133b-3p miR-30d-5p	miRNA	Osteoblasts	N/A	RUNX2	Inhibiting osteoblast differentiation	[108,109]
**In Osteoarthritis**						
circ_0001846	circRNA	Human chondrocyte cells	Increase	miR-149–5p/ WNT5B axis	Modulating IL-1β-induced chondrocyte cell damage	[242]
circ-BRWD1	circRNA	Human chondrocyte cells	N/A	miR-1277/ TRAF6 axis	Contributing to OA development	[243]
circRNA_0001236	circRNA	MSCs	Increase	miR-3677-3p/Sox9 axis	Enhancing chondrogenesis and suppressing cartilage degradation	[244]
lncRNA H19	lncRNA	The fibroblast-like synoviocyte	Decrease	miR-106b-5p/ TIMP2 axis	Inhibiting the degradation of the matrix in osteoarthritis	[245]
miR-8485	miRNA	Chondrocytes	N/A	Wnt/β-catenin, GSK-3β	Stimulating the cartilage differentiation of BMSCs	[246]
miR-9-5p	miRNA	BMSCs	N/A	SDC1	Reducing inflammation and OA-like injury	[247]
miR-26a-5p	miRNA	hBMSCs	Increase	PTGS2	Delay synovial fibroblast damage in vitro and reduce OA damage	[248]
miR-320c	miRNA	hBMSCs	Increase	N/A	Promoting the proliferation of hBMSC chondrocytes and downregulating matrix metallopeptidase 13	[249]
miR-100-5p	miRNA	Human exfoliated deciduous teeth	Increase	mTOR-3′ untranslated region	Inhibiting the inflammation of temporomandibular joint (TMJ) chondrocytes	[250]
miR-100-5p	miRNA	IPFP-MSCs	Increase	mTOR	Promoting the abnormal gait of OA mice and reducing the pathological changes of articular cartilage in vivo	[251]
miR-135b	miRNA	MSCs	Increase	Sp1	Promoting chondrocyte proliferation, thereby promoting cartilage repair	[252]
miR-92a-3p	miRNA	MSCs chondrocyte	Increase	WNT5A	Promoting cartilage proliferation and matrix gene expression in MSCs	[253]
miR-95-5p	miRNA	Primary chondrocytes	Increase	HDAC2/8	Regulated cartilage development and homogenous balance by direct targeting HDAC2/8	[254]
lncRNA PCGEM1	lncRNA	Synovial fluid	Increase	N/A	Exosomal lncRNA PCGEM1 may be a novel indicator to distinguish early OA from late OA	[255]
lncRNA KLF3-AS1	lncRNA	MSCs	Increase	miR-206/GIT1 axis	Promoting the expression of GIT and alleviating the chondrocyte damage induced by IL-1β	[256]
lncRNA KLF3-AS1	lncRNA	MSCs	Increase	Col2a1	Inhibiting IL-1β-induced chondrocyte apoptosis	[257]
miR-140-5p	miRNA	Human synovial MSCs	Increase	N/A	Promoting cartilage regeneration and delaying the progression of knee OA	[258]
miR-185-5p miR-7107-5p	miRNA	Synovial fluid	N/A	TLR signaling pathway	Suppress chondrocyte/ chondrogenesis; promote inflammation	[259]
lncRNA HULC	lncRNA	Chondrocytes	Increase	N/A	Promoting cell apoptosis and inhibiting cell proliferation	[260]
miR-193b	miRNA	Plasma	Decrease	HDAC3	Promoting histone H3 acetylation and regulating the metabolism of primary human chondrocytes	[261]
miR-200C	miRNA	Synovial fluid	Increase	N/A	miR-200C increased 2.5 times in OA exosomes compared with non-OA patients	[262]
**In Bone Fracture Repairing**						
miR-5106	miRNA	M1D	Increase	SIK2	Inducing osteogenic differentiation of BMSCs	[263]
miR-126	miRNA	MSCs	Decrease	HIF-1α	Promoting bone fracture repairing/healing	[264]
miR-128-3p	miRNA	MSCs	N/A	SMAD 5	Regulate bone formation and fracture healing	[265]
LncRNA-MALAT1	lncRNA	Endothelial progenitors	N/A	miRNA-124	Leading to bone repair	[266]
miR-125b-5p miR-338-3p miR-21 miR-4532	miRNA	MSCs	Increase	N/A	May help to enhance bone formation and angiogenesis	[267]

### 8.4. Treatment of Osteoarthritis by Exsomal miRNAs

OA is bone degeneration in joints that causes cartilage degeneration, synovitis, chronic pain, and disability. OA is characterized by extracellular matrix (ECM) loss and cartilage destruction; treatment is focused on attenuating pain symptoms [268]. In OP progression, OA may be treated by joint-replacement surgery. However, complete repair or regeneration of damaged articular cartilage is difficult [269]. Exosomal miR-26a-5p derived from hBMSC are highly expressed in OA, and inhibit synovial fibroblast damage and prostaglandin-endoperoxide synthase-2, which are significant in OA treatment (Table 1) [248]. Existing evidence suggests that TGF-β1 regulates Sp1 through MSC-derived exosomal miR-135b, promoting chondrocyte proliferation and cartilage repair [252]. Interestingly, using exosomal miR-92a-3p from MSCs enhances the expression of ECM genes in MSCs and promotes cartilage proliferation [253]. The gene histone deacetylase (HDAC) is targeted by miR-193b, supporting histone (H3) acetylation and directing the metabolism of primary human chondrocytes [261]. Interleukin-6 (IL-6)-mediated inflammation is inhibited by miR200C. In studies, exosomal miR-200C expression in synovial fluid of OA patients was found to increase by 2.5 times more than in healthy individuals [102,262]. Moreover, miR-4454 is associated with the inflammatory response, and miR-199b is involved in cartilage formation. Researchers have examined 50 miRNAs in the exosomes of synovial fibroblasts with differential expression [270,271]. 

Several inflammatory factors are linked to the stimulation of exosomal ncRNAs that regulate OA. A pathway, HDAC2/8, is involved in the inhibition of cartilage development through cartilage-specific genes. However, miR-95-5p in primary chondrocytes regulates cartilage development through HDAC2/8 [254,272]. Overexpression of miR-100-5p has been identified in stem cells that are linked with temporomandibular joint (TMJ) inflammation. Upregulation of miR expression may be associated with the occurrence and progression of OA [102,273]. 

Experimentally, a BMSC-derived exosomal miR-9-5p injection reduces inflammation and OA-like injury in the mouse. In one study, exosomal miR-9-5p targeting the syndecan-1 gene, upregulation led to an exacerbation of inflammation and OA damage [247]. Exosome-like vesicles from chondrocytes of OA patients were shown to stimulate inflammation and increase the production of mature IL-1β by macrophages through the miR-449a-5p/ATG4B/autophagy pathway, thereby aggravating synovitis and accelerating OA progression [274]. Chondrocytic exosomal miR-8485 stimulates cartilage differentiation in BMSCs by promoting the Wnt/β-catenin pathway [246]. 

## 9. Concluding Remarks and Future Prospectives

Conclusively, several non-coding miRNAs, including miR-290-295, miR-302, miR-17-92, miR-106b-25, and miR-106a-363, and lncRNAs, including H19, HOTAIR, lncRNA-ES1, lncRNA-ES3, and lncRNA-ES2, are differentially expressed in ESCs and efficiently control the self-renewal and pluripotency status of stem cells. The miRNAs miR-134, miR-296, and miR-470 are functionally upregulated to suppress negative regulators and to enhance pluripotent transcription factors such as mouse genes Nanog, Oct4, *c-Myc*, and Sox2 in an epigenetic manner in ESCs. The 3′UTR regions of mRNAs are the principal recipients of miRNA activity. However, silent mutations in the predicted target interfere with miRNA activity and inhibit the downregulation of corresponding genes. The deregulated cell cycle is linked to tumorigenesis, which is further linked to metastasis, invasion, and therapeutic resistance. Although ncRNAs also act as tumor suppressor genes targeting oncogenic pathways, including miRlet-7, miR-31, miR-34, and miR-17-92, these are associated with the prognosis of cancer patients. Hence, the ncRNA-based bone disease treatment strategy can be highly significant, as it interferes with cell cycle abnormalities within the disease microenvironment. NcRNAs inhibit or promote cell cycle regulators and modulate disease development in diseases including OP, OA, and bone tumors. Despite the different therapeutic applications, ncRNAs studies will greatly encourage the understanding of bone health as well as its disease status.

## Data Availability

Not applicable.
